# Effects of temperature and intrinsic structural defects on mechanical properties and thermal conductivities of InSe monolayers

**DOI:** 10.1038/s41598-020-72162-9

**Published:** 2020-09-15

**Authors:** Van-Trung Pham, Te-Hua Fang

**Affiliations:** 1 Department of Mechanical Engineering, National Kaohsiung University of Science and Technology, Kaohsiung, 807 Taiwan; 2 Institute of Research and Development, Duy Tan University, Danang, 550000 Vietnam

**Keywords:** Materials science, Nanoscience and technology

## Abstract

We conduct molecular dynamics simulations to study the mechanical and thermal properties of monolayer indium selenide (InSe) sheets. The influences of temperature, intrinsic structural defect on the tensile properties were assessed by tensile strength, fracture strain, and Young’s modulus. We found that the tensile strength, fracture strain, and Young’s modulus reduce as increasing temperature. The results also indicate that with the existence of defects, the stress is concentrated at the region around the vacancy leading to the easier destruction. Therefore, the mechanical properties were considerably decreased with intrinsic structural defects. Moreover, Young’s modulus is isotropy in both zigzag and armchair directions. The point defect almost has no influence on Young’s modulus but it strongly influences the ultimate strength and fracture strain. Besides, the effects of temperature, length size, vacancy defect on thermal conductivity (*κ*) of monolayer InSe sheets were also studied by using none-equilibrium molecular dynamics simulations. The *κ* significantly arises as increasing the length of InSe sheets. The *κ* of monolayer InSe with infinite length at 300 K in armchair direction is 46.18 W/m K, while in zigzag direction is 45.87 W/m K. The difference of κ values in both directions is very small, indicating the isotropic properties in thermal conduction of this material. The *κ* decrease as increasing the temperature. The κ goes down with the number of atoms vacancy defect increases.

## Introduction

After graphene was first prepared by Novoselov et al.^1^ in 2004, two-dimensional (2D) materials have been attracted a lot of attention due to their remarkable and wide potential applications such as energy conversion, energy storage, electronics, optoelectronics, and thermal management^2–6^. Recently, inspired by the investigation of graphene, other 2D materials such as borophene^7^, phosphorene^8^, silicene^9^, germanene^10^, MoS_2_^11^ have also been studied by numerous researchers. A new 2D materials called indium selenide (InSe), an III–VI group van der Waals layered structures materials, has been obtaining much consideration due to its outstanding optical and electronic properties. InSe belongs to the family of metal chalcogenide layer semiconductors^12,13^. The monolayer InSe consists of four covalently bonded Se–In–In–Se sublayers. Recently, this material has received much attention. For instance, several experimental works have demonstrated that several atomic layers of InSe can be fabricated^14–16^. Zhou et al.^17^ shown the successful synthesis of monolayer InSe by the physical vapor deposition (PVD) method. Chang et al.^18^ synthesized InSe atomic layers by vapor phase selenization of In_2_O_3_ in a chemical vapor deposition (CVD) system. Hung et al.^19^ evaluated the thermoelectric properties of monolayer InSe, while Jalilian et al.^20^ investigated the structural, electronic, and optical properties of InSe under tensile and compressive strain condition.

Numerous researchers have reported that InSe has efficient applications on nano-devices. For example, Tamalapudi et al.^21^ have demonstrated that the devices of few-layer InSe have an extremely strong photoresponse with superior photoresponsivities to those of other lately reported 2D crystal-based (GaS, GaSe, MoS_2_, and graphene) photodetectors. Balakrishnan et al.^22^ have presented that the technological potential of mechanically formed heterojunctions and homojunctions of direct-bandgap layered GaSe and InSe compounds with an optical response over an expanded wavelength range, from the near-infrared to the visible spectrum. These researchers have been shown that monolayer InSe is a promising material for optoelectronics and photosensitive devices. In addition, 2D InSe engages for the application of bendable photodetectors with broadband response^23^, 2D ferromagnets^24^, and topological insulator^25^. The adsorption-induced magnetic behaviors on InSe monolayer was also reported on some studies^26,27^.

In recent year, we found that most studies have been conducted based on the first-principles calculation methods. There was only Chang et al.^28^ used the molecular dynamics (MD) simulation to study the mechanical properties of single layer InSe under axial tension. The first-principles calculation methods are famous to provide relatively accurate results, but these approaches are limited to small structures due to high computation requirements^29^. As an alternative approach, MD simulation can be utilized to analyze very large systems, with thousands to millions of atoms^30^. Additionally, as a complement to the experimental test, MD simulation is a helpful tool to deepen the understanding of the mechanical and thermal properties at the nanoscale^31–35^.

The above articles have mainly researched with perfect InSe monolayer. However, experimentally fabricated InSe monolayer is not generally perfect and may exist in different types of defects. During fabrication, native defects, impurities, and extrinsic dopants are inevitably or purposefully introduced. Compared to bulk materials, 2D material defects tend to have a more significant influence on their material properties^36,37^. However, the mechanical and thermal properties of the defective InSe monolayers have not been explored yet. Motivated by this, we performed MD simulations to study the effect of temperature and intrinsic structural defects on the mechanical and thermal properties of InSe monolayers. The fracture behavior, Young’s modulus, tensile strength, and fracture strain are discussed in the presence of various intrinsic vacancy defects. We also surveyed the effect of temperature and intrinsic structural defects on the deformation behavior under tensile loading along various directions. Moreover, the impacts of length size, temperature, and defects on the thermal properties of InSe sheets were also studied. For the evaluation of thermal conductivity (*κ*) of monolayer InSe sheets, none-equilibrium molecular dynamics (NEMD) approach was used.

## Results

### Effect of temperature on the mechanical characteristics of monolayer InSe

In this section, we investigated the influence of temperature on the deformation and fracture process of single layer InSe during uniaxial tension in the both armchair and zigzag directions. Moreover, we also investigated the tensile strength, fracture strain, and Young’s modulus affected by temperature.

**Figure 1:**
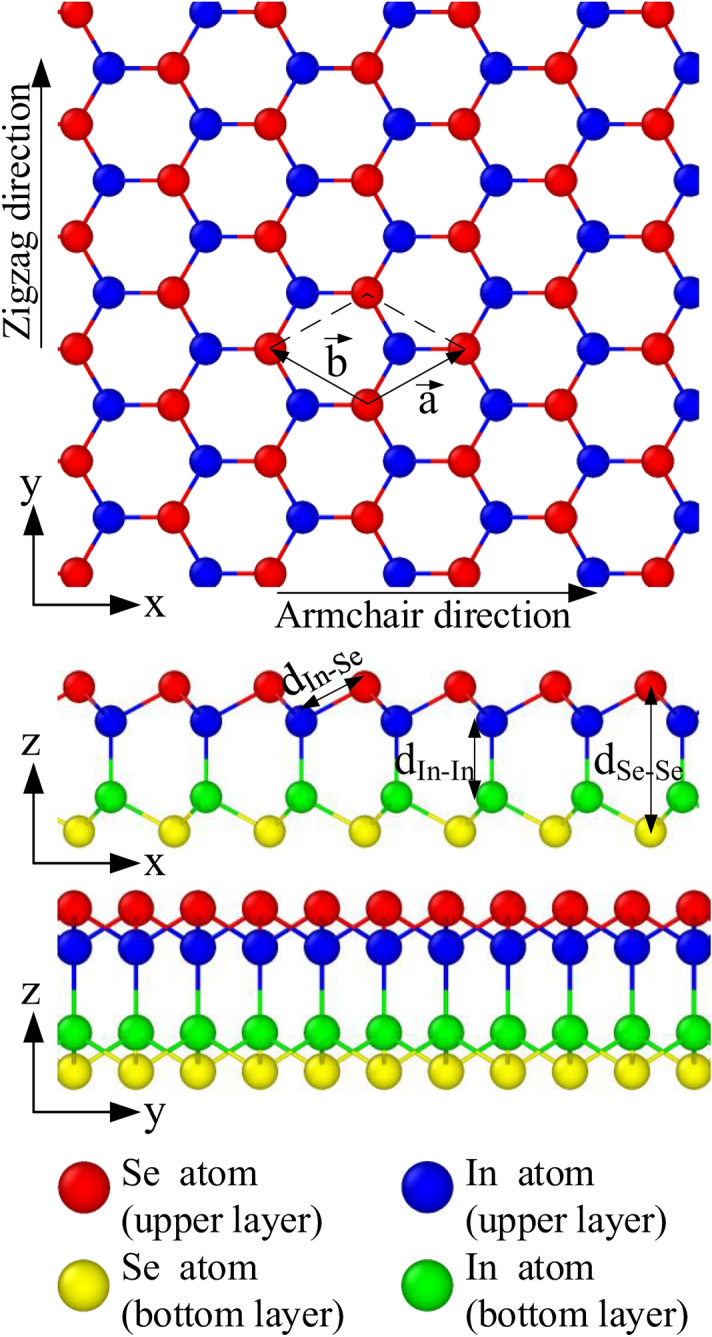
Atomic configuration of monolayer InSe.

Figure 1 shows the atomic structure of monolayer InSe to study the mechanical properties under tensile process. The dimension of the sample is 200 Å × 200 Å along the *x-* (armchair) and *y-* (zigzag) directions. The periodic boundary conditions were applied in three directions to eliminate the finite-length effect. A vacuum layer is 50 Å along the *z*-direction between a monolayer and its periodic image so that the atom will not interact across the periodic boundary in this direction. The bucking distance and the lattice parameters of InSe structure are: a = b = 4.093 Å, d_In–In_ = 2.816 Å, d_Se–Se_ = 5.385 Å, d_In–Se_ = 2.689 Å which are consistent with first-principles calculation^38^. Similar to reported of Shafique et al.^39^, we use d_Se–Se_ = 5.385 Å as the thickness in the thermal conductivity calculation.

**Figure 2:**
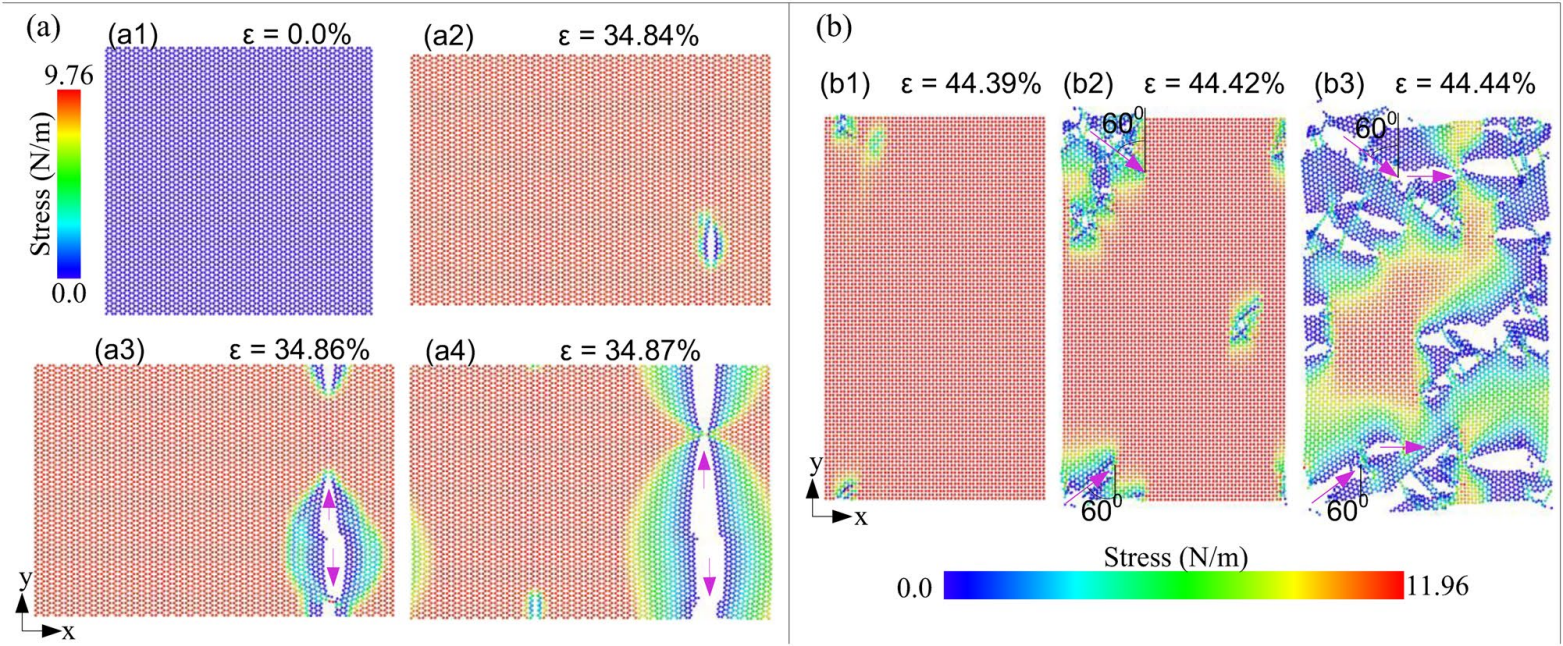
Deformation and fracture process of single layer InSe during uniaxial tensile at 1 K (**a**) along the x (armchair) direction and (**b**) along the y (zigzag) direction.

Figure 2 shows a series of snapshots of the deformation and fracture process of single layer InSe during uniaxial tensile at 1 K along the *x* (armchair) direction and along the *y* (zigzag) direction. It is evident that as the strain grows from 0 to 34.84%, the stress rises from 0.0 to 9.76 N/m in Fig. 2a1,a2. At the strain of 38.84%, the sheet starts to fracture. After that, the crack spreads perpendicular to the tensile direction, and as the strain increase to 34.87% the sheet was fractured completely. For the tensile process along the *y* (zigzag) direction at 1 K, the fracture processes of single layer InSe were illustrated in Fig. 2b. The stress increases from 0.0 to 11.96 N/m as the strain increases from 0 to 44.39%. A small fracture is observed in the nanosheet at the strain of 44.39%. As increasing the strain, the fracture process increases, and the sheet is completely broken at the strain of 44.44%. Interestingly, under different tensile directions, the fracture surfaces are distinctively different. For the single-layer InSe sheet under uniaxial tension in the armchair direction, a smooth fracture surface and the crack is perpendicular to the tension direction. Whereas, a relatively rough fracture surface is inspected when the sheet is stretched along the zigzag direction. Firstly, some cracks appear at the strain of 44.39% and the propagation direction of the crack is at a 60° to the loading direction. The cracks continue to propagate and when the strain reaches 44.43% the direction of the cracks becomes perpendicular to the tensile direction, as presented in Fig. 2b3. Consequence, the fracture deformation prefers to spread along the zigzag edge for both loading cases, suggesting that the zigzag direction has smaller surface energy than the armchair direction. This result is similar to the previous result of Wang et al.^40^, which studied the fracture mechanics of monolayer molybdenum disulfide. This result is also good agreement with Chung et al.^41^ and Sun et al.^42^, which investigated the tensile behaviour of graphene.

**Figure 3:**
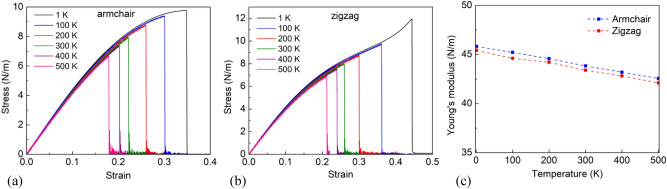
Tensile properties of the InSe nanosheet with different loading temperatures. (**a**) Stress–strain curves of the InSe nanosheet subjected to uniaxial tension along the armchair direction. (**b**) Stress–strain curves of the InSe nanosheet subjected to uniaxial tension along the zigzag direction. (**c**) Young’s modulus.

We studied the effect of temperature on the mechanical properties of single layer InSe by performing tension processes with the temperature ranging from 1 to 500 K. Figure 3 shows the tensile properties of the InSe nanosheet with different loading temperatures. At 1 K, the tensile stress and the ultimate strain in the armchair direction are 9.76 N/m and 0.3484, while the fracture stress and the ultimate strain in the zigzag direction are 11.96 N/m and 0.4439. These results are consistent with the report of Chang et al.^28^. We can see that both the tensile strength and the fracture strain reduce as raising the temperature. Into specifics, as the temperature rises from 1 to 500 K, the tensile strength and fracture strain drop by 31.50% and 48.65% under the tensile process in the armchair direction. Correspondingly, in the zigzag direction, the tensile strength and fracture strain drop by 41.79% and 52.29%. This temperature-induced softening phenomenon is explained by our previous reports^43,44^: the atomic thermal vibrations get more dramatically robust and the vibrational amplitudes seem to be more tremendous while the temperature is growing up. This growing prompts the interatomic distance expands, as a result, a decrease of the bond energy of the atoms in the sheet, which results in the chemical bonds are more easily achieve the critical bond length and crack. Consequently, the tensile strength and fracture strain decrease as the temperature increases. To more understand the effect of temperature on the mechanical characteristics, Young’s modulus of single layer InSe sheet was also determined. From Fig. 3a,b, Young’s modulus of single layer InSe was calculated as the function of temperature, as demonstrated in Fig. 3c. The Young’s moduli of monolayer InSe at 1.0 K in the armchair direction determined is 45.83 N/m and in the zigzag direction is 45.42 N/m. This result is similar to the first-principles calculation results in Hu et al.^38^. Young’s modulus reduces as growing the temperature. At 500 K, Young’s modulus in the armchair direction is 42.56 N/m and it is 42.11 N/m in the zigzag direction, the reduction is about 7.14% and 7.29% comparing with those of 1 K, respectively. Therefore, Young’s modulus is not as sensitive to temperature as the fracture strength. Additional, with the same temperature the different of Young’s modulus in the tension along the armchair direction and the zigzag direction is very small, so Young’s modulus of the single layer InSe is isotropy. This result is confirmed with reported by Chang et al.^28^ and Hu et al.^38^.

The comparison of tensile strength, fracture strain, and Young’s modulus of single layer InSe in this research with some other 2D materials is summarized in Table 1. It is evident that, the results of this work are very close to the results in previous studies. This study provides useful knowledge and can be used as a reference for further studies about monolayer InSe in the future. From this table, it is observed that Young modulus of InSe is lower than that of graphene, borophene. The fracture strain of single layer InSe is higher than that of graphene, phosphorene, borophene, which suggests that single layer InSe exhibits notable flexibility.

**Table 1:** Comparison of the tensile mechanical properties of monolayer InSe.

Material	Young’s modulus (N/m)	Tensile strength (N/m)	Fracture strain (%)	Temperature (K)	References
InSe	44.8 (zigzag)	12.0	0.44	1	Chang et al.^28^
	45.7 (armchair)	9.8	0.35		
InSe	45.61 (zigzag)	5.2	0.25	0	Hu et al.^38^
	45.61 (armchair)	5.9	0.27		
InSe	45.42 (zigzag)	11.96	0.4437	1	This research
	45.83 (armchair)	9.76	0.3481	1	
Graphene	350	39.5	0.2	0	Liu et al.^45^
Phosphorene	92.01 (zigzag)	10.0	0.30	1	Hu et al.^46^
	22.15 (armchair)	4.0	0.27		
Borophene	162.49 (zigzag)	18.48	0.3	0	Wang et al.^47^
	378.97 (armchair)	12.26	0.12		

### Effect of defects on the mechanical properties

**Figure 4:**
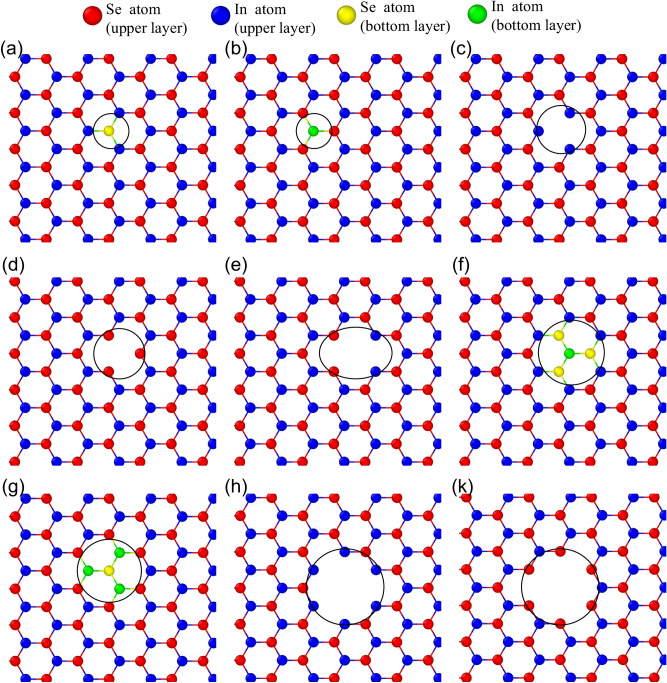
The point defects in monolayer InSe. The black circles illustrate the position of defects. Defects are called (**a**) single selenide vacancy (VSe1), (**b**) single indium vacancy (VIn1), (**c**) diselenide vacancy (VSe2), (**d**) diindium vacancy (VIn2), (**e**) vacancy of double InSe pair (VInSe2), (**f**) vacancy of In and nearby three Se atoms (V3Se1In), (**g**) vacancy of Se and nearby three In atoms (V1Se3In), (**h**) vacancy complex of In and nearby three Se pairs (V6Se2In), (**k**) vacancy complex of Se and nearby three In pairs (V2Se6In).

The influence of defects on the mechanical properties was investigated in this section. Detailed lists of intrinsic structural defects used in this study are provided in Fig. 4. As a typical example, we consider InSe with a supercell having 200 Å × 200 Å size. The defects location is located at the center of the example. We implement periodic boundary conditions along three directions to avoid any edge effects.

**Figure 5:**
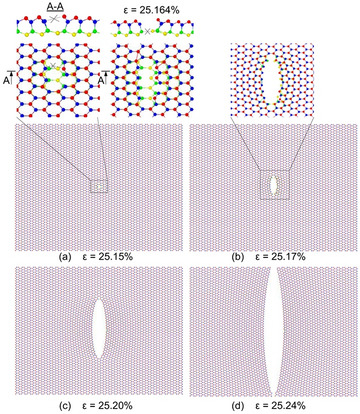
Deformation and fracture process of single layer InSe with single Se defect during uniaxial tensile along the armchair direction at 1 K.

**Figure 6:**
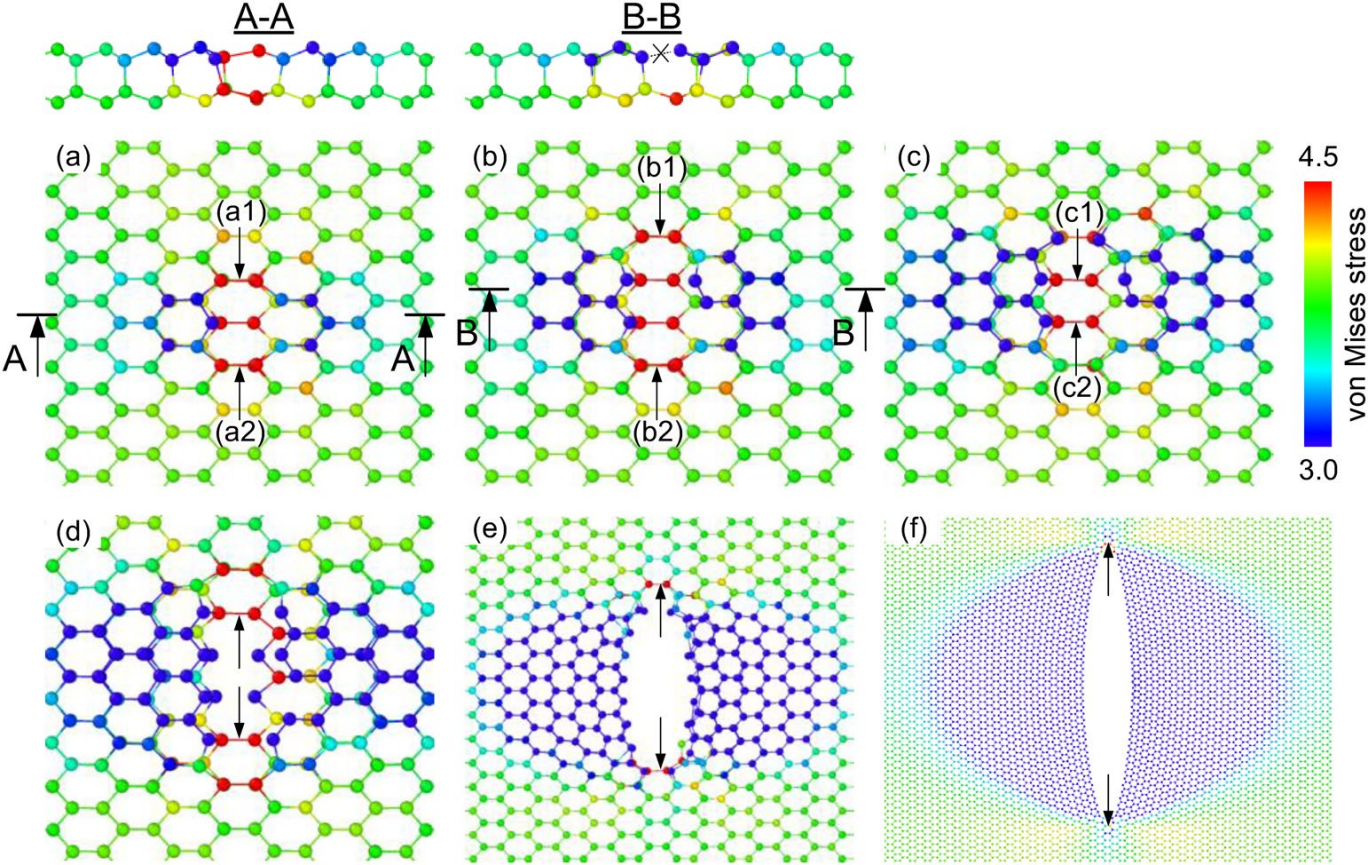
The von Mises stress distribution of the defective InSe sheet at the strain of (**a**) 25.150%, (**b**) 25.155%, (**c**) 25.162%, (**d**) 25.164%, (**e**) 25.168%, (**f**) 25.220%. In atoms and Se atoms are colored according to their atomic stress.

Figure 5 depicts the deformation and fracture process of single layer InSe with single Se defect during uniaxial tensile in the armchair direction at 1.0 K. Comparing with the perfect sheet, the destruction of the defective sheet occurs easier. At the strain of 25.151%, the bond of In–Se atoms besides the defect begins breaking, as shown in section A–A. At the strain of 25.164%, the bond of In–Se atoms on the bottom layer, which besides the defect begins breaking. With further increases of the strain, the crack develops quickly in the direction perpendicular to the tensile direction until the InSe sheet is completely broken. To explain the decrease in strength of the defect sheet compared to the perfect one, we use the atomic stress distribution as shown in Fig. 6.

Figure 6 shows the von Mises stress distribution of the defective InSe sheet under the tensile process in the armchair direction. It can be seen that the stress concentration occurs at the region around the vacancy, resulting in the bonds in this area more susceptible to fracture, leading to the crack nucleation. At the strain of 25.150% in Fig. 6a, we note that the maximum stress is located at (a1), and (a2) bonds where are most likely to rupture. When the strain raises to 25.155% in Fig. 6b, one of these most dangerous bonds is ruptured, shown in section B–B. Then the maximum of stress is concentrated at the new position, at (b1) and (b2) bonds. When the strain increases to 25.162% in Fig. 6c, the (b1) bond is broken, and then the maximum of stress is concentrated at (c1) and (c2) bonds. When the strain raises to 25.164%, the (c1) and (c2) bonds were ruptured. The crack grows as increasing the tensile strain, and this process ultimately results in the failure of the InSe sheet. This concentration of stress makes the fracture process easier and leads to reduced tensile strength.

Similar with the single Se defect, the deformation and fraction process of other kinds of defect occurs easily than the perfect sheet, and significant stress concentration is also focus around the defect, that makes the crack initiates at the vacancy. To further explore the influences of the kind of defects on the mechanical properties, we also simulated models with different defects under the tensile process along the armchair direction and zigzag direction; the corresponding results are presented in Supplementary Figs. 1 and 2.

Supplementary Fig. 1 illustrates the stress–strain relations of the single layer InSe at 1.0 K, for various vacancy defects under uniaxial tension in the armchair direction. Overall, the vacancy defect has a strong effect on the stress–strain curves. With the vacancy defects at the In atoms position, the mechanical properties of InSe sheets are more influenced than that sheets with the vacancy defects at Se atoms position. For example, VIn affect the mechanical properties more than VSe, V1Se3In affect the mechanical characteristics more than V3Se1In, V2Se6In affect the mechanical characteristics more than V6Se2In. With the larger vacancies, the stress–strain curves are more affected, such as V2Se6In and V6Se2In affect the mechanical properties more than V1Se3In and V3Se1In, V2In affect the mechanical properties more than V1In, V3Se1In affect the mechanical properties more than V1In, and so on. Completely similar to the analysis above, the tension process along the zigzag direction also shows that defects affect stress–strain curves, as shown in Supplementary Fig. 2.

**Figure 7:**
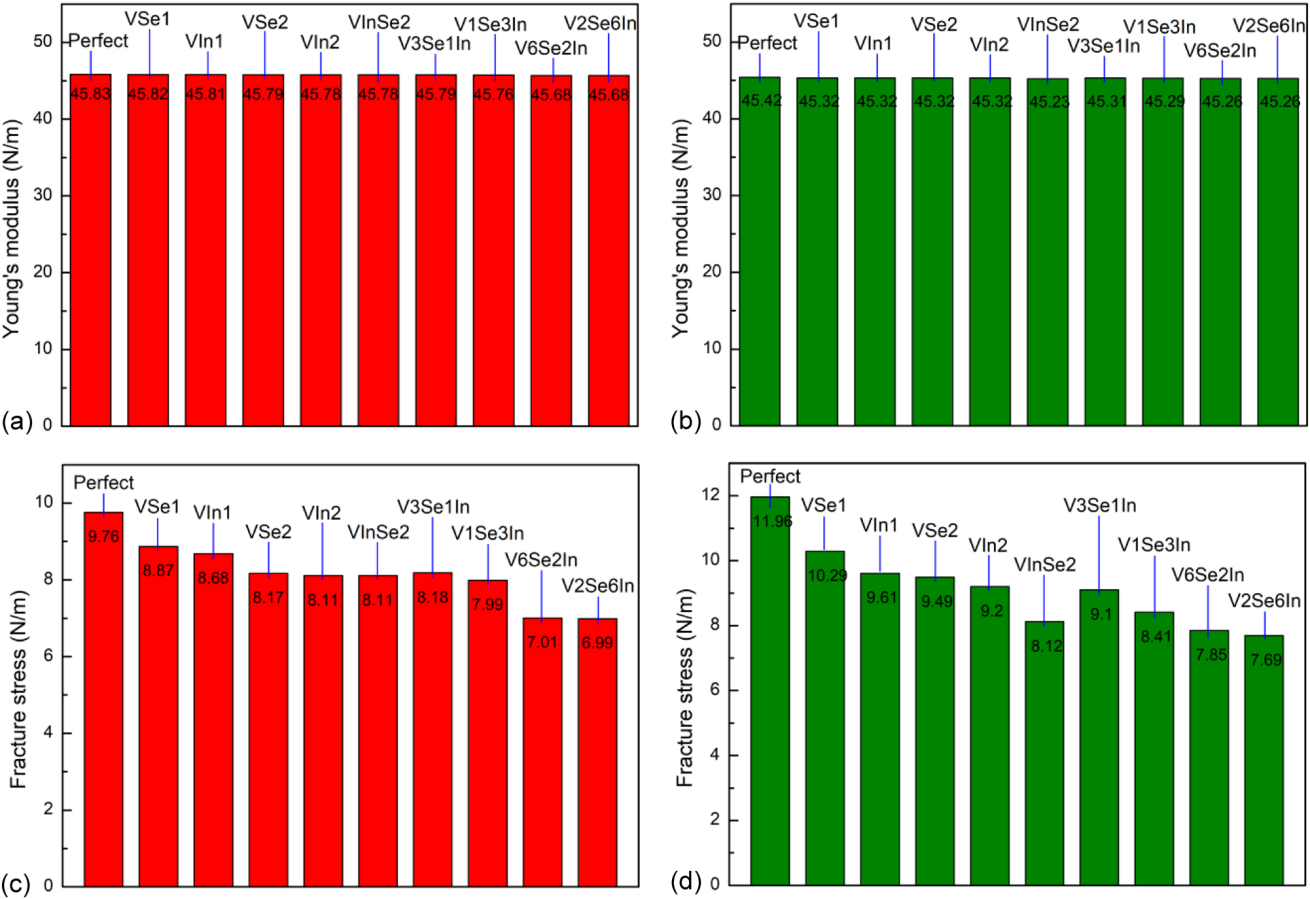
The effect of defects on the tensile properties of the monolayer InSe at 1.0 K. (**a**) Young’s modulus under uniaxial tension along the armchair direction. (**b**) Young’s modulus under uniaxial tension along the armchair direction. (**c**) Tensile strength under uniaxial tension along the armchair direction. (**d**) Tensile strength under uniaxial tension along the zigzag direction.

Figure 7 shows the effect of defects on the tensile properties of the monolayer InSe at 1.0 K. We can see that with different kinds of point defects, Young’s modulus has minor affected by point defect. With the same defect, Young’s modulus value in the tension along the armchair direction is very close with its value in the zigzag direction; hence, Young’s modulus is isotropy. Figure 7c,d illustrates the effect of different vacancy defects on tensile strength under uniaxial tension in various directions. The results present that the tensile strength goes down with the number of atoms vacancy increases. It also indicated the negative influence of vacancy defects on the mechanical properties. It is interesting to see that the point defect almost has no influence on Young’s modulus, but it is very sensitive to the tensile strength and fracture strain. The missing In or Se atoms fractures the perfect bonding in the monolayer InSe, and that makes stress concentrate around the vacancy defect. Consequently, the crack is easily nucleating around the vacancy and results in the tensile strength and fracture strain greatly decrease.

In fracture mechanics, one parameter used to determine the fracture of a single layer InSe sheet is the critical stress-intensity factor (SIF). We use the critical stress-intensity factor *K*_*IC*_ to express the combination of the effects of the stress at the crack tip and the crack length. The subscript *I* indicates mode *I* testing in which a tensile stress causes the crack to open. The critical SIF can be estimated from the following equation^48^:

1 $$ K_{IC} = \sigma_{f} \sqrt {\pi a} $$

where *σ*_*f*_ is the fracture stress; *a* is half the length of an internal through crack.

The vacancy defects V6Se2In can be seen as an internal crack, and the schematic illustration of pre-cracked InSe sheets under tension is shown in Supplementary Fig. 3. From the Supplementary Fig. 1, the fracture stress value of InSe sheet with V6Se2In defect under uniaxial tension along the armchair direction is 7.01 N/m. Then, the critical SIF *K*_*IC*_*t* determined is 3.08 × 10^–4^ N/m^1/2^ under tension in the armchair direction. Similarly, the critical SIF *K*_*IC*_*t* determined is 3.39 × 10^–4^ N/m^1/2^ under tension in the zigzag direction, where *t* is the thickness of monolayer InSe.

### Effect of system size on the thermal conductivity

**Figure 8:**
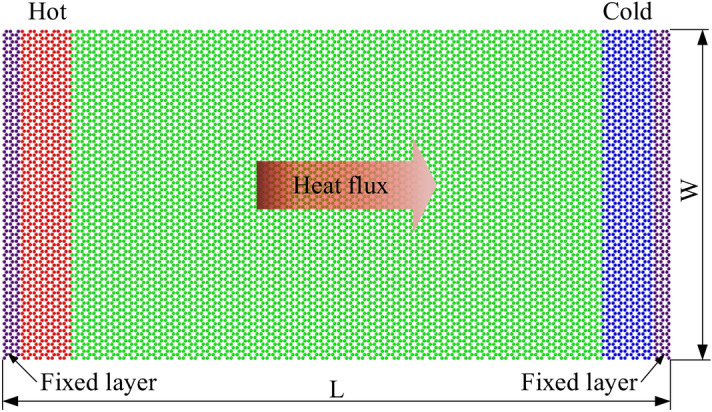
Schematics of the non-equilibrium MD simulation of monolayer InSe.

We used the model in Fig. 8 to compute the thermal conductivity. Figure 8 presents the MD model for the evaluation of thermal conductivity with a size of *L*  ×  *W*, where *L* is the length of the sample, *W* is the width of the sample. To explore the effect of sample length on the thermal conductivity, we conducted various MD simulations with different lengths from 20 to 160 nm along the heat flux direction and with a fixed width of 20 nm. Because periodic boundary condition was used in the width direction so the width size did not affect the calculation results, which demonstrated by Hong et al.^49^ and Liang et al.^50^. Therefore, the width of 20 nm is applied in all systems for thermal conductivity computation.

**Figure 9:**
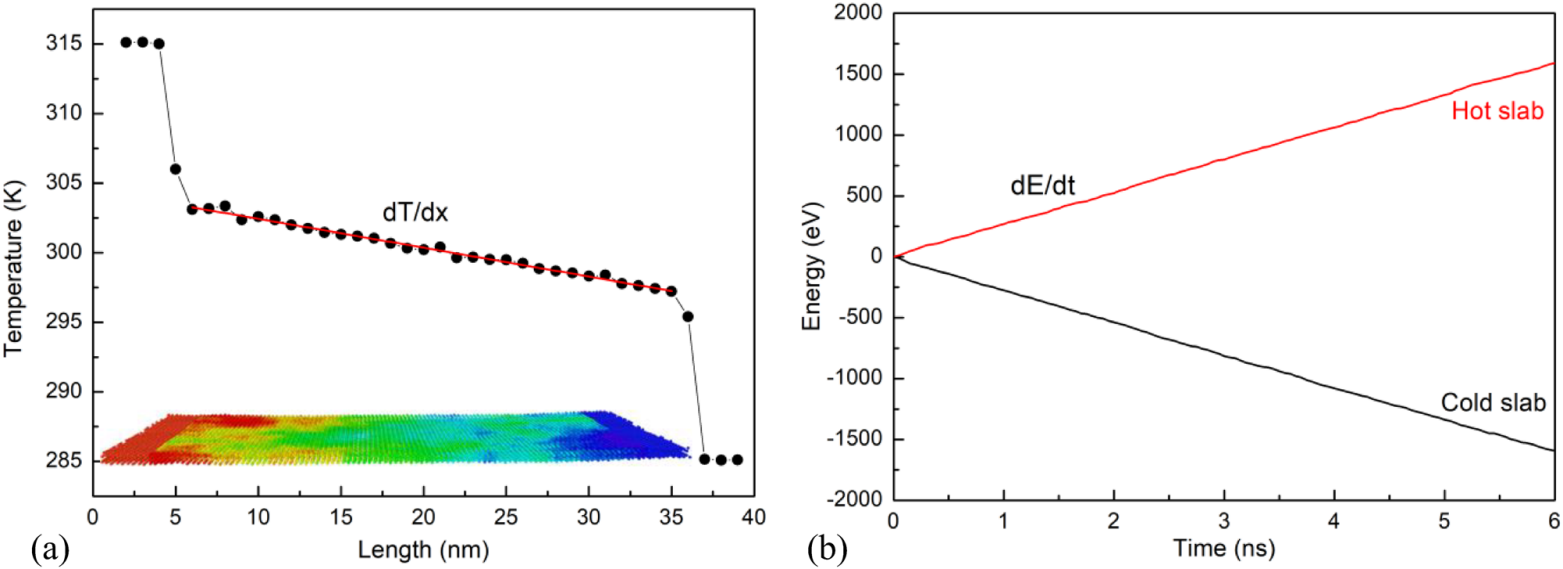
(**a**) Relationship between temperature distribution and position of slabs for armchair at 300 K with L = 40 nm. (**b**) Energies added to the hot slab and removed from the cold slab with respect to the time.

Figure 9a presents the relationship between temperature distribution and position of slabs for armchair at 300 K and L = 40 nm. The temperature distribution is a large fluctuation in the hot and cold areas. Other regions have small fluctuations due to a phonon scattering phenomenon^51^. The temperature gradient is the function of the atom position along the heat flux transfer direction. In this figure, the red line denotes for the linear fitting of the temperature profile, giving the temperature gradient *dT*/*dx*. Figure 9b illustrates the energies added to the hot slab and removed from the cold slab with respect to the time. As it is illustrated, the amount of energy added to the hot slab is equal to the amount of energy removed from the cold slab, which implies the total energy of the system is conserved. Furthermore, we can see that the slopes of energies are linear which means that the energies added to the hot slab and removed from the cold slab at a constant rate. This result is similar to the previous study of Mortazavi et al.^52^, which investigated the thermal conductivity of defective graphene. We used the expression (10) in the Methods section to determine the thermal conductivity, the *κ* calculated is 18.37 (W/m K) and 18.16 (W/m K) for the monolayer InSe along the armchair direction and zigzag direction, respectively. The difference of *κ* along both directions is very small, so the monolayer InSe is isotropy in thermal conductivity, which is in good agreement with the first-principle calculation by Nissimagoudar et al.^53^.

**Figure 10:**
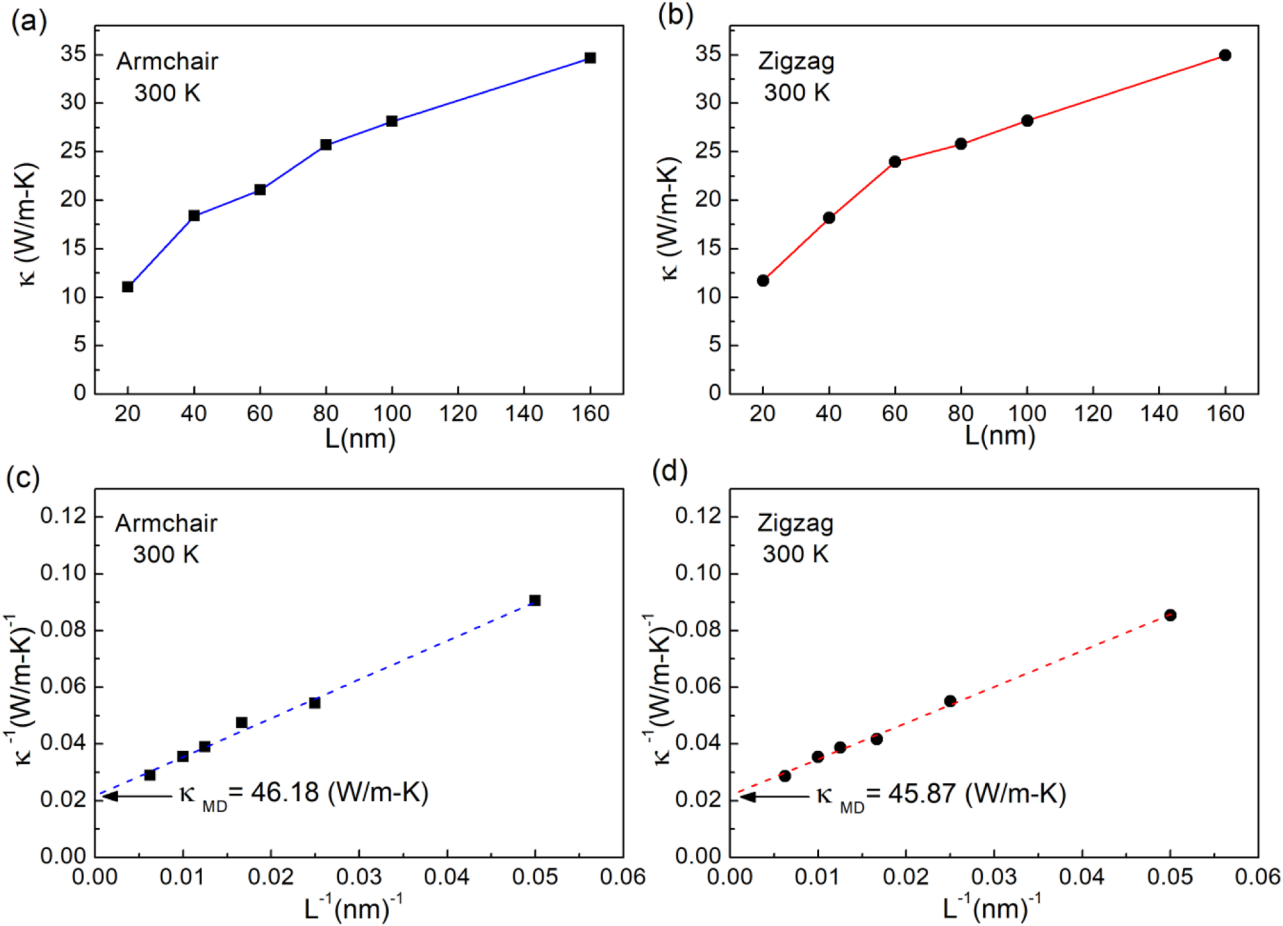
Length dependence of thermal conductivities for monolayer InSe in (**a**) armchair direction and (**b**) zigzag direction. Linear fitting of 1/κ and 1/L for (**c**) armchair direction and (**d**) zigzag direction.

Figure 10 presents the thermal conductivity as a function of sheet length at temperature 300 K in (a) armchair direction and (b) zigzag direction. Moreover, it also illustrates the linear fitting of 1/κ and 1/L for (c) armchair direction and (d) zigzag direction. From our NEMD simulations data, we see that the size affect strongly to *κ.* The *κ* values with the length of 20.0, 40.0, 60.0, 80.0, 100.0 and 160.0 nm in armchair direction are 11.05, 18.37, 21.04, 25.71, 28.14, and 34.68 W/m K, respectively. While the *κ* values with the length of 20.0, 40.0, 60.0, 80.0, 100.0, and 160.0 nm in zigzag direction are 11.70, 18.16, 23.98, 25.80, 28.22, and 34.95 W/m K, respectively. The *κ* increases with increasing sample size due to the participation of a larger subset of phonon in heat transfer, it is similar to reported in Ref.^53,54^. Therefore, to obtain the material-intrinsic (i.e. size-independent) thermal conductivity of monolayer InSe systems, we used the extrapolating formula following^55,56^.

2 $$ \frac{1}{\kappa (L)} = \frac{1}{{\kappa_{\infty } }}\left( {\frac{l}{L} + 1} \right) $$

where κ_∞_ is the intrinsic thermal conductivity of an infinitely length monolayer (*L → ∞*), *l* is effective phonon mean free path, and *L* is the length of the system.

The fitting results of *1*/*κ* with *1*/*L* for armchair and zigzag direction are given in Fig. 10c,d, respectively. By linear extrapolating to 1/L = 0, the thermal conductivity of infinitely length InSe in the armchair direction is 46.18 W/m K, whereas in zigzag direction is 45.87 W/m K. The comparison of the thermal conductivity in this study to some other papers is provided in Table 2. In thermal conductivity calculation, the thickness of 2D materials directly affects the numerical results. Nissimagoudar et al.^53^ used 8.32 Å as the thickness of monolayer InSe, so they found the thermal conductivity to be 27.6 W/m K. Whereas, this study uses the thickness of 5.385 Å, which is close to the thickness values used in the calculation of Shafique et al.^39^,Wan et al.^57^, and Pandey et al.^58^. Therefore, it has been found that these simulation results are consistent with the first-principles calculations. From this table, it is observed that thermal conductivity of InSe is lower than that of graphene, phosphorene. The thermal conductivity of InSe is on the same magnitude as that of borophene^60^, however, the Young’s modulus of InSe is lower than that of borophene significantly^47^. The results implied InSe possesses the weaker phononic anharmonicity than borophene. This is significant information for designing of thermal management devices.

**Table 2:** Comparison of the thermal conductivity of monolayer InSe.

2D material	*κ* (W/m K)	Temperature (K)	Method	References
InSe	44.4	300	First-principles	Shafique et al.^39^
InSe	27.60	300	First-principles	Nissimagoudar et al.^53^
InSe	44.60	300	First-principles	Wan et al.^57^
InSe	41.67	300	First-principles	Pandey et al.^58^
InSe	46.18 (armchair)	300	Classical MD simulation	This research
	45.87 (zigzag)			
Graphene	1,008.5 (armchair)	300	Classical MD simulation	Hong et al.^59^
	1,086.9 (zigzag)			
Phosphorene	63.6 (armchair)	300	Classical MD simulation	Hong et al.^59^
	110.7 (zigzag)	300		

### Effect of temperature on the thermal conductivity

To explore the effect of temperature on the thermal conductivity, we conducted various MD simulations with different temperatures from 100 to 500 K for the armchair direction and zigzag direction with the sample dimensions of 20 × 20 nm^2^.

**Figure 11:**
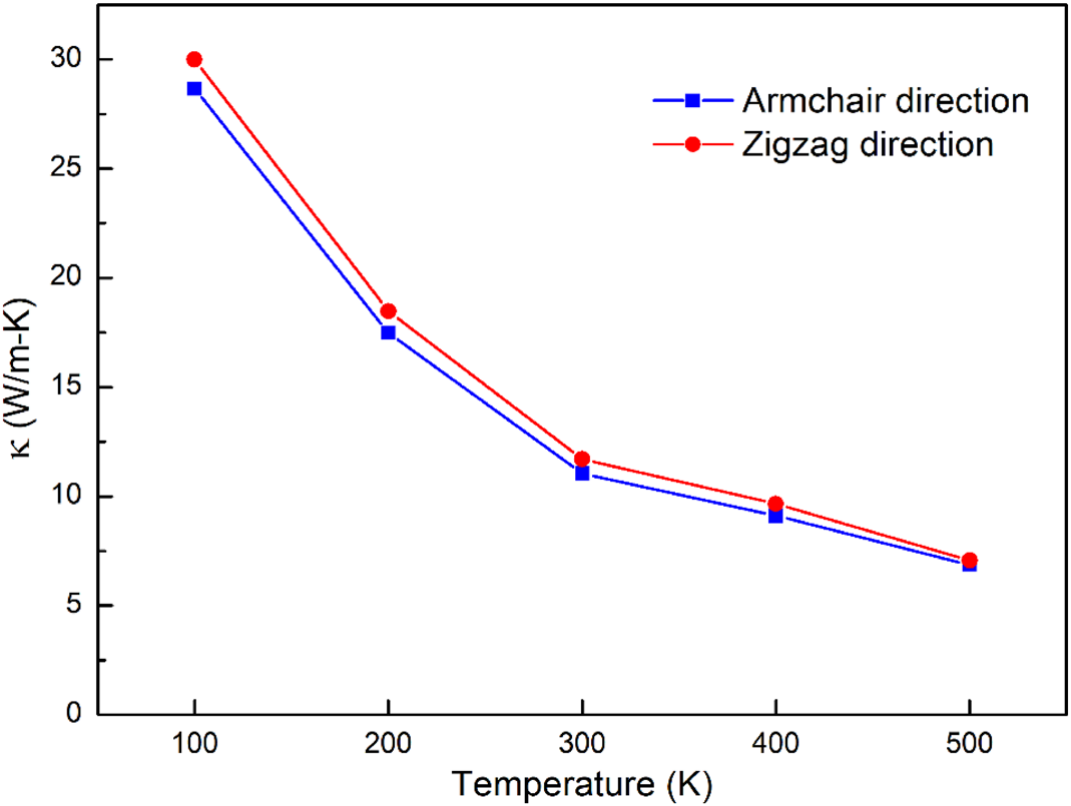
Relationship between thermal conductivity and temperature with the sample dimensions of 20 × 20 nm^2^.

Figure 11 illustrates the relationship between thermal conductivity and temperature for the armchair direction, and for the zigzag direction. The *κ* values with a temperature of 100, 200, 300, 400, and 500 K in armchair direction are obtained 28.67, 17.48, 11.05, 9.12, and 6.86 W/m K, respectively. While the *κ* values with a temperature of 100, 200, 300, 400, and 500 K in zigzag direction are obtained 29.98, 18.47, 11.70, 9.66, and 7.07 W/m K, respectively. The thermal conductivity decrease as increasing the temperature. This phenomenon can be explained by Umklapp phonon–phonon scattering. High temperature leads to larger momenta of phonon. When these phonons with large momenta clashed with other phonons with high momenta, lead to losing their energy since the total resulting momenta easily exceeds the allowed momentum in the lattice. Therefore, at higher temperatures, Umklapp phonon scattering becomes more serious; hence making the excited phonon modes to be offset at high temperatures, lead to the thermal conductivity decrease. This result is similar to the study of Park et al.^61^.

### Effect of intrinsic structural defects on the thermal conductivity

The influence of intrinsic structural defects on the thermal conductivity was investigated in this section. Detailed lists of intrinsic structural defects used in this study are provided in Fig. 4. The defects location is located at the center of the example. The relationship between temperature distribution with position of slabs, and the temperature profile of the InSe sheet with V2Se6In defect is presented in the Supplementary Fig. 4. We can see that the temperature is continuous. With another kind of defect, the results are similar.

**Figure 12:**
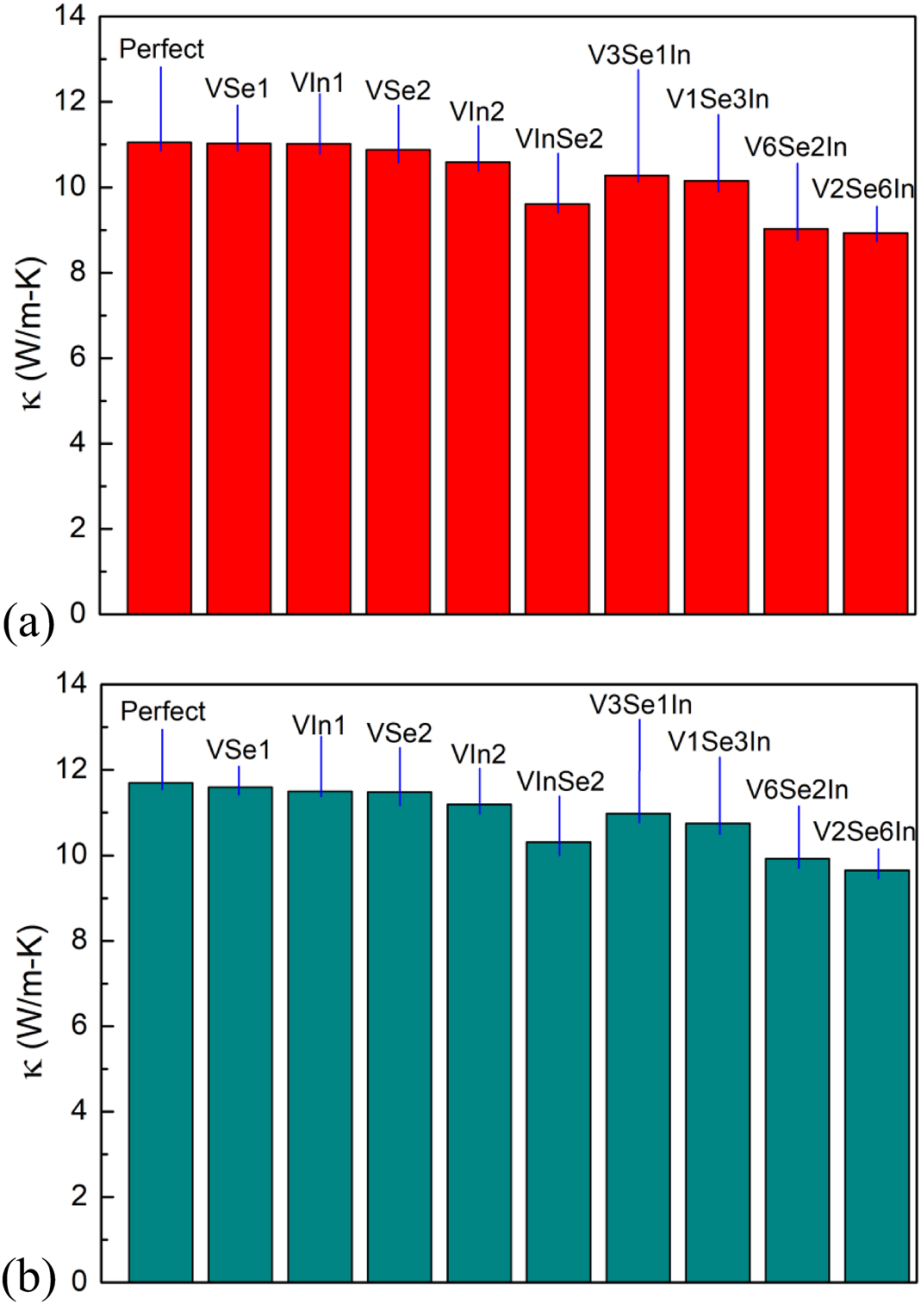
Relationship between thermal conductivity and intrinsic structural defect at 300 K with the sample dimensions of 20 × 20 nm^2^ for (**a**) armchair direction and (**b**) zigzag direction.

Figure 12 shows the relationship between thermal conductivity and intrinsic structural defect at 300 K with the sample dimensions of 20 × 20 nm^2^ for armchair direction (a) and zigzag direction (b). The results illustrate κ goes down with the number of atoms vacancy increases, it also indicated the negative effect of vacancy defects on the thermal conduction. It is interesting to see that the In defects are more influential than the Se defects such as V2Se6In affect the thermal conduction more than V6Se2In. This phenomenon due to the scattering of phonons at the defect centers, leading to the reduction in phonon mean free path. To further understand the thermal transport mechanism for defective InSe sheets, we analyzed contributions of phonon scattering to the thermal resistance. According to the theory of classical lattice thermal conductivity, the thermal conductivity is proportional to the mean free path for phonon scattering by the following expression^62^:

3 $$ \kappa = CVl $$

where *C* is the specific heat capacity; *V* is the phonon group velocity of the sound wave in solid; *l* is the phonon mean free path. With the defective InSe sheets, in addition to phonon–phonon scattering, we should consider the scattering at the defects. In the presence of defects, the phonon mean free path is changed to:

4 $$ \frac{1}{l} = \frac{1}{{l_{phonon - phonon} }} + \frac{1}{{l_{phonon - defect} }} $$

where *l*_*phonon–phonon*_ is the phonon–phonon scattering length, and *l*_*phonon-defect*_ denotes the phonon-defect scattering length. According to Wang and Tabarraei^62^, defect can induce considerable phonon-defect scattering which decreases the phonon mean free path. In view of Eqs. (3) and (4), we can get the relation

5 $$ \frac{1}{\kappa } \propto \frac{1}{{l_{phonon - phonon} }} + \frac{1}{{l_{phonon - defect} }} $$

It is clearly indicated in Eq. (4) that the phonon-defect scattering leads to decreasing in the phonon mean path. According to Zhang et al.^63^, as increasing the defect size leads to phonon-defect increase, and *l*_*phonon-defect*_ decrease, thus a reduction in the thermal conductivity according to Eq. (5). These MD simulation results are consistent with discovered by other researchers^64,65^.

## Discussion

In conclusion, the tensile response and thermal conductivity of monolayer InSe were investigated through MD simulations. For the tensile properties, the effect of temperature, intrinsic structural defects were investigated. The results indicate that the tensile strength, fracture strain, and Young’s modulus decrease as the temperature increases. With the same defect, Young’s modulus in the tension along the armchair direction is very close in the tension along the zigzag direction, thus Young’s modulus is isotropy. Interestingly, the point defect almost has no influence on Young’s modulus but it is very sensitive to the tensile strength and fracture strain. The missing In or Se atoms fractures the perfect bonding in the monolayer InSe, and that makes stress concentrate around the vacancy defect. Consequently, the crack is easily nucleating around the vacancy and results in the tensile strength and fracture strain greatly decrease.

Besides that, for the thermal properties, the effect of system lengths, temperature, and intrinsic structural defects were also studied. The obtained results show that MD simulations, in excellent agreement with first-principles calculations. The *κ* of monolayer InSe at 300 K in armchair direction is 46.18 W/m K, while in zigzag direction is 45.87 W/m K. The difference of κ along both directions is very small, so the monolayer InSe sheet is isotropy in thermal conductivity. The thermal conductivity decrease as increasing the temperature. The κ goes down with the number of atoms vacancy increases, indicating the negative effect of vacancy defects on the thermal conduction.

## Methods

### MD simulations for tension process

In this research, all MD simulations are performed using the Large Scale Atomic/Molecular Massively Parallel Simulator (LAMMPS) package^66^. The visualization is realized using the open visualization tool (OVITO)^67^. The recently Stillinger–Weber (SW) potential parameterized by Jiang et al.^68^ is used for modeling the interactions between In, Se atoms in the InSe system.

Before tension, we adopt the conjugate gradient method to relax the systems. Then the sample was equilibrated under the isothermal-isobaric (NPT) ensemble at zero pressure and the specified temperature for 100 ps with a time step of 0.5 fs. After that, the uniaxial tensile strains with a constant engineered strain rate of 10^–4^ ps^–1^ were applied in either armchair or zigzag direction. The tensile deformations were also simulated in the NPT ensemble until complete fracture took place. The simulations were conducted at a different temperature ranging from 1 to 500 K and the temperatures were controlled by using the Nosé–Hoove method.

During the tension deformation, the strain is determined using the following equation:

6 $$ \varepsilon = \left( {L - L_{0} } \right)/L_{0} $$

where the initial and final length of the model along the tension direction are expressed by L_0_ and L, respectively. For further investigate stress distribution of the sheets under tensile processes, the atomic stress of the monolayer InSe during the uniaxial tension was calculated by the viral theorem using equation^69,70^:

7 $$ \sigma_{ab} = \frac{1}{V}\left( { - \sum\limits_{i} {m_{i} \nu_{ia} \nu_{ib} } + \frac{1}{2}\sum\limits_{i} {\sum\limits_{i \ne j} {r_{ijb} F_{ija} } } } \right) $$

where *V* is the volume of the system, the indices *a* and *b* denote the Cartesian components. *m*_*i*_ and *v*_*i*_ are the atomic mass and velocity of the particle *i*, respectively, *F*_*ij*_ is the interatomic force, and *r*_*ij*_ denotes the distance between atoms *i* and *j*.

The thickness of mono-atomic crystal structures such as graphyne, graphene, and carbon nanotubes or InSe cannot be easily determined exactly^71^. Due to the ambiguity for the thickness of those 2D materials, Cranford et al.^72^ suggested the stress and elastic moduli of the monolayer system be reported in force per unit length (N/m) rather than force per unit area (N/m^2^ or Pa). Then, N/m is the unit of atomic stress in this study.

The von Mises stress *σ*_*von*_ is defined as follows^70^:

8 $$ \sigma_{von}^{2} = \frac{1}{2}\left[ {\left( {\sigma_{xx} - \sigma_{yy} } \right)^{2} + \left( {\sigma_{yy} - \sigma_{zz} } \right)^{2} + \left( {\sigma_{zz} - \sigma_{xx} } \right)^{2} + 6\left( {\sigma_{xy}^{2} + \sigma_{yz}^{2} + \sigma_{zx}^{2} } \right)} \right] $$

### Thermal conductivity calculations

Free boundary conditions are employed in the out-of-plane direction (*z*) and the heat flux direction (*x*) while periodic boundary condition is applied in the width direction (*y*).

Before applying the heat flux in the system, an energy minimization was conducted using the conjugate gradient algorithm. The equations of atomic motion are integrated with a time step of 0.5 fs. Then the thermal equilibrium is established in the system at a prescribed temperature under a canonical NVT ensemble and coupling to Nosé–Hoover temperature thermostat for 10^6^ time steps. Then, the system is switched to an NVE (constant number of atom, volume, energy) ensemble for 10^6^ time steps to conserve the energy and volume of the system. Thus, the system is equilibrated and the system is ready for heat flux processing. After the system is equilibrated, the system also keeps in the NVE ensemble while the NEMD approach is employed to calculate the thermal conductivity. The sample was divided into 40 slabs along the heat transfer direction. The regions near the left/right end were treated as the hot/cold slabs, the temperature of which is set to (T + ΔT) K at the hot slab and (T – ΔT) K at the cold slab, respectively, where ΔT = 5% of T. The non-equilibrium steady-state heat flux J was created by using the Langevin thermostat for 12 × 10^6^ time steps. When the system can reach the steady-state, the heat flux J can be expressed by:

9 $$ J = \frac{dE/dt}{A} $$

where A is the cross-section area of the simulation system in the direction perpendicular to the heat transfer, E is the accumulated energy, t is the simulation time.

Once the heat flux is imposed, the temperature gradient is obtained, the thermal conductivity k of the system is determined by using the Fourier law:

10 $$ k = \frac{J}{dT/dx} $$

In this paper, the temperature at each slab is given by^73,74^:

11 $$ T{}_{k} = \frac{1}{{3n_{k} k_{B} }}\sum\limits_{i \in k}^{{n_{k} }} {m_{i} v_{i}^{2} } $$

where *T*_*k*_ is the temperature of *k*th slab, *n*_*k*_ is the number of atoms in slab *k*, *m*_*i*_ and *v*_*i*_ are the mass and velocity of atom *i*, *k*_*B*_ is Boltzmann’s constant.

## Supplementary information

Supplementary Information.
