# Supplementary material for: Effects of temperature and intrinsic structural defects on mechanical properties and thermal conductivities of InSe monolayers
Source: Sci Rep. 2020 Sep 15;10:15082. doi: 10.1038/s41598-020-72162-9 (PMC7492280; doi:10.1038/s41598-020-72162-9)
Supplement: Supplementary file 1 — Supplementary Information. [file 41598_2020_72162_MOESM1_ESM.docx]

Supplementary Information for

**Effects of temperature and intrinsic structural defects on mechanical properties and thermal conductivities of InSe monolayers**

Van-Trung Pham^1,2^ and Te-Hua Fang^1,*^

^1^Department of Mechanical Engineering, National Kaohsiung University of Science and Technology, Kaohsiung 807, Taiwan

^2^Institute of Research and Development, Duy Tan University, Danang 550000, Vietnam

^*^Corresponding author. Email address: [fang@nkust.edu.tw](mailto:fang@nkust.edu.tw) (T.H. Fang)


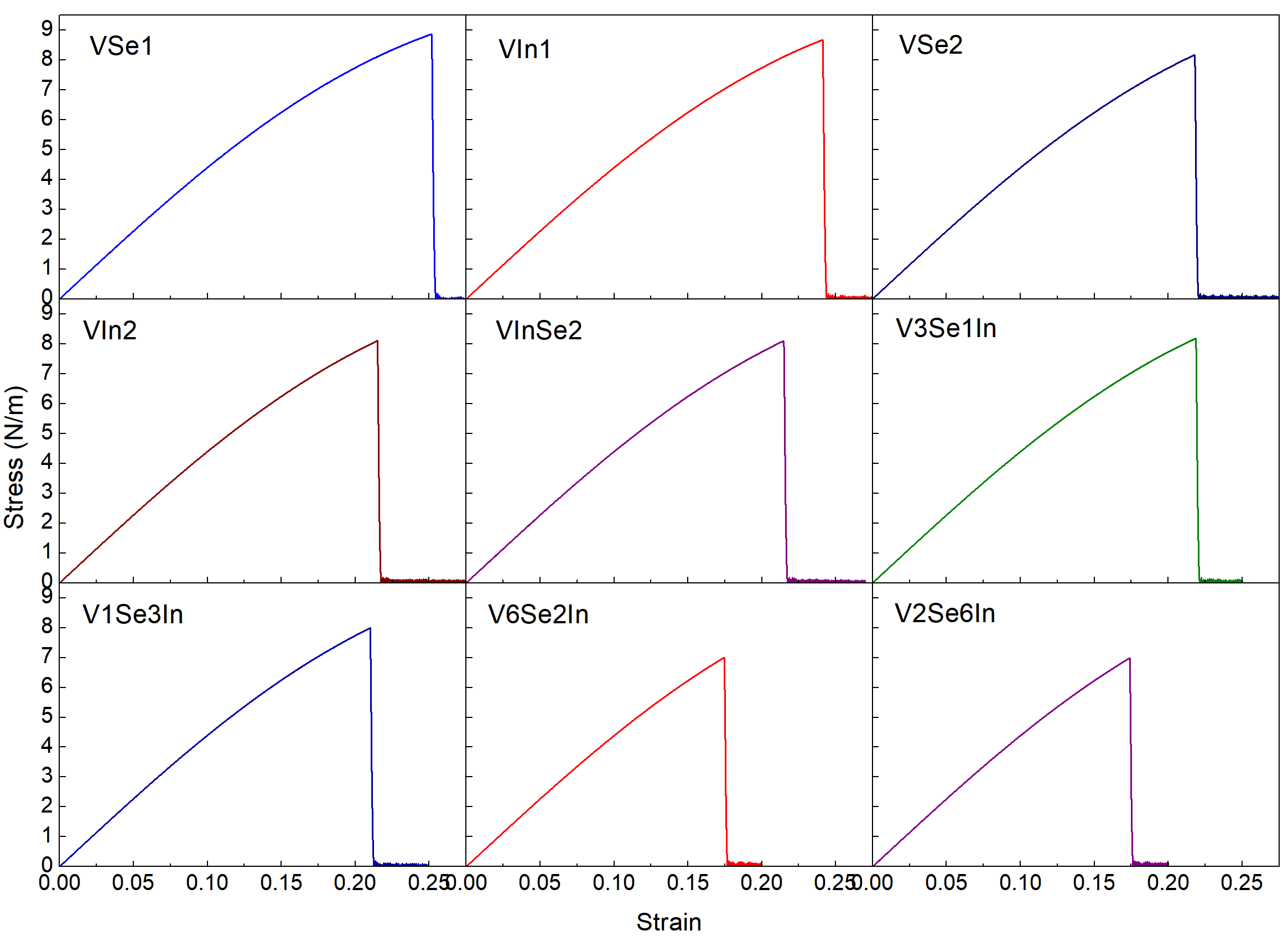


**Supplementary Fig. 1.** Stress-strain relations of the InSe nanosheet at 1 K, for different vacancy defects under uniaxial tensile along the armchair direction.

**
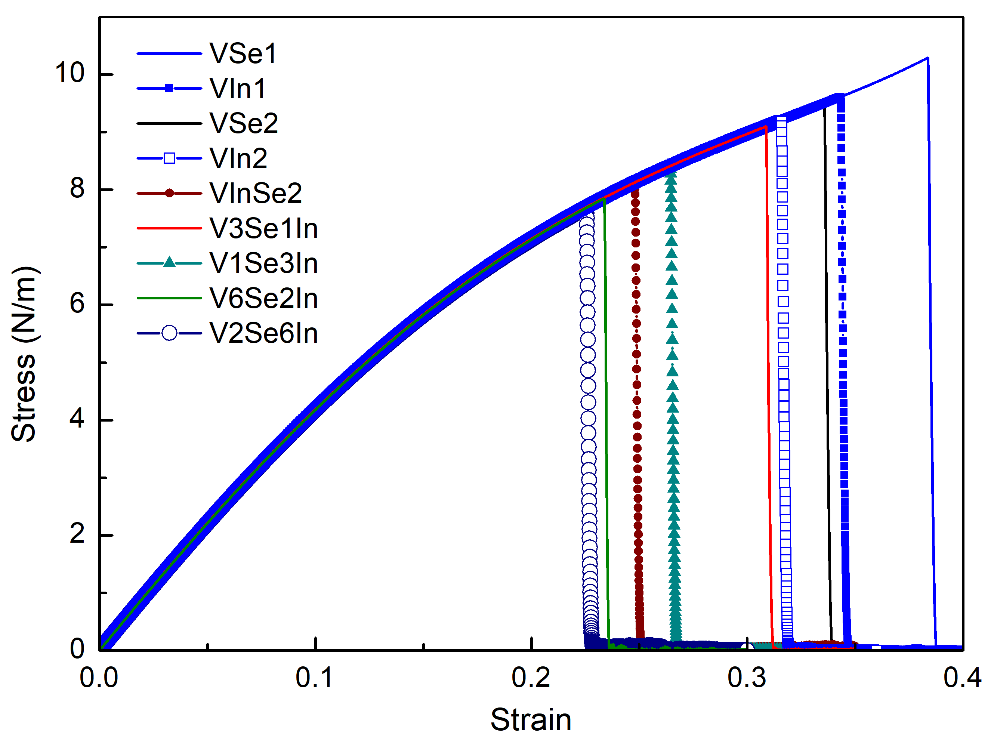
**

**Supplementary Fig. 2.** Stress-strain relations of the InSe nanosheet at 1 K, for different vacancy defects under uniaxial tensile along the zigzag direction.

**Supplementary Fig. 3.** Schematic illustration of pre-cracked InSe sheets under tension: (a) in the armchair direction, (b) in the zigzag direction.

**Supplementary Fig. 4.** (a) Relationship between temperature distribution and position of slabs of the InSe sheet with V2Se6In defect for armchair at 300 K with L = 20 nm. (b) The temperature profile of the InSe sheet with V2Se6In defect for armchair at 300 K with L = 20 nm.
